# Production of Cellulosic Microfibers from Coffee Pulp via Alkaline Treatment, Bleaching and Acid Hydrolysis

**DOI:** 10.3390/ma16247607

**Published:** 2023-12-12

**Authors:** Eliud S. Rodriguez-Quiroz, Octavio Olivares-Xometl, Verónica Santacruz-Vázquez, Claudia Santacruz-Vázquez, Paulina Arellanes-Lozada, Efraín Rubio-Rosas

**Affiliations:** 1 Facultad de Ingeniería Química, Benemérita Universidad Autónoma de Puebla, Av. San Claudio y Blvd. 18 sur, Puebla 72570, Mexico; eliudsalvador.rodriguez@alumno.buap.mx (E.S.R.-Q.); octavio.olivares@correo.buap.mx (O.O.-X.); veronica.santacruz@correo.buap.mx (V.S.-V.); 2 Centro Universitario de Vinculación y Transferencia de Tecnología, Benemérita Universidad Autónoma de Puebla, Prol. 24 sur y Av. San Claudio, Puebla 72570, Mexico; efrain.rubio@correo.buap.mx

**Keywords:** lignocellulosic compounds, biomass, coffee pulp, microcellulosic fibers, delignification

## Abstract

The present work deals with the production of cellulosic microfibers (CMFs) from coffee pulp. The experimental development corresponds to an experimental design of three variables (concentration, temperature and time) of alkaline treatment for delignification, finding that concentration, temperature and time were the most representative variables. Higher delignification was achieved by bleaching cellulosic fibers, followed by acid hydrolysis, thus producing cellulosic fibers with an average diameter of 5.2 µm, which was confirmed using scanning electron microscopy-energy-dispersive X-ray spectroscopy (SEM-EDS). An X-ray diffraction (XRD) analysis revealed, via the crystallinity index, the presence of Type I cellulose and removal of lignocellulosic compounds through chemical treatments. The proximate chemical analysis (PChA) of coffee pulp helped to identify 17% of the crude fiber corresponding to the plant cell wall consisting of lignocellulosic compounds. The initial cellulose content of 26.06% increased gradually to 48.74% with the alkaline treatment, to 57.5% with bleaching, and to 64.7% with acid hydrolysis. These results attested to the rich cellulosic content in the coffee pulp.

## 1. Introduction

In the last decade, this approach has encouraged a growing necessity to conceive and obtain “green chemistry” materials due to their compatibility with the environment and high biodegradability [1,2]. An example of green material is the cellulosic fiber present in plant lignocellulosic compounds [3]. Since cellulose is omnipresent in all plant structures, it is a low-cost-abundant raw material [4]. Properties such as biocompatibility, hydrophilicity and non-toxicity, among others, have set cellulose as a natural source matter with high potential for different applications like reinforcing material in a number of polymeric matrix composites [5,6,7,8]. In this context, coffee pulp is a solid organic residue with a high cellulose content [9,10,11]. In the coffee cherry, 55% of its content corresponds to the seeds, which are employed as a raw material for human consumption, and the remaining 45% is represented by solid organic residue [12]. According to the Food and Agriculture Organization of the United Nations (FAO), the generation of coffee pulp worldwide is, on average, 6,198,286.07 tons yearly and its acquisition does not represent any economic cost; furthermore, the inadequate handling of this residue is becoming a socioenvironmental problem.

On the other hand, cellulosic fibers are constituted by straight carbohydrate polymer chains consisting of β-(1→4)-linked glucopyranose units and have a polymerization degree of about 10,000 [13]. In addition, they display a semi-crystalline structure with morphology and dimensions that depend on the raw matter [14]. Cellulose produced by plants has two different forms of crystalline cellulose (types I and II) and is classified in four separate polymorphs [15]. The isolation of cellulose from crude plant fiber involves the separation of the lignocellulosic compounds (lignin, hemicellulose and cellulose); however, there is a problem regarding recalcitrance, for there is a hierarchy from the crystalline cellulose surrounded by lignin incrustation in the hemicellulose matrix, which are compounds bound by covalent and non-covalent bonds [16]. There are physical treatments that diminish the polymerization degree by reducing the size by means of mechanical actions such as grinding, extrusion and ultrasound, which in addition have the advantage of being environmentally friendly, but with high energy costs [17]. Chemical treatments through alkaline and acid hydrolysis are more efficient and the control of the conditions and operation variables is simple. Notwithstanding, the suitable selection of alkaline and acid reagents to carry out the hydrolysis is required, for it has been evidenced that weak acids are not that effective [18]. In contrast, biological treatments employ microorganisms that consume lignin and hemicellulose, and for this reason, they are the most ecological, but the control of the treatment conditions lowers the efficiency of cellulose production [19]. As for ionic liquids (ILs), they are relatively new methods and the solubilization of the lignocellulosic compounds is achieved by their ions; nevertheless, these treatments are expensive and toxic when combined with enzymes [3,20]. There are also treatments known as organosolv that use organic solvents which provoke the breaking of internal bonds between lignin and hemicellulose, but the process scaling increases the costs. In addition, the inflammability and volatility features demand specially controlled operation conditions [19,21]. Finally, physicochemical treatments with ammonia, CO_2_ and steam explosion employ mechanical forces such as temperature or high pressure, which produce an explosion that helps to break the cellulose and hemicellulose glycosidic bonds; unfortunately, these treatments demand high amounts of energy and money [17,22].

Additionally, through the production of cellulosic microfibrils by employing a combination of different treatments and methods, high yields have been reported. Nakagaito and Yano studied the benefit of the properties of cellulose microfibrils obtained via the microfibrillation of kraft pulp fiber through mechanical refining processes and high-pressure homogenization [23]. In another study carried out by Bahloul et al., cellulose microfibers with fiber diameters between 2 and 50 μm and consisting of amorphous and crystalline areas, whose production depended on the source and isolating conditions, were obtained by employing alkaline and bleaching treatments [24]. In this context, Soykeabkaew et al. worked on starch-based biocompounds reinforced with jute and bacterial cellulose obtained via film casting, emphasizing that the reinforcement in the composites is essentially affected by the fiber nature and load amount [25]. Due to the points mentioned above, it is necessary to keep on looking for treatments that allow the synthesis of microcellulosic fibers from cellulose-rich biomass and whose production costs and environmental impacts are both minimal [3,26]. To this end, the present work aims to develop green materials represented by microcellulosic fibers by employing organic waste from the coffee industry, which is easily available due to its high production. A proximate chemical analysis (PChA) was performed to establish the crude fiber content of coffee pulp. Physical, alkaline, bleaching and acid hydrolysis treatments were combined to produce CMFs. In addition, the lignin, hemicellulose and coffee pulp cellulose contents were obtained from the American Society for Testing and Materials (ASTM). Finally, the delignification process was carried out and assessed by the crystallinity index, employing the XRD technique and being reinforced by SEM-EDS.

## 2. Materials and Methods

### 2.1. Materials

Coffee pulp (skin of the coffee green beans) is a residue produced by the mechanical processes of threshing and depulping that are employed to obtain the green seeds destined for consumption [27]. In the present study, the raw material was coffee pulp obtained from coffee fields located in the Cuetzalan municipality, Puebla, Mexico. This material was employed to carry out the PChA and extraction of cellulosic fibers. As for the PChA, H_2_SO_4_ (95% purity, Wöhler, Mexico City, Mexico) and NaOH (97% purity, Meyer, Mexico City, Mexico) were the chemical reagents. The determination of the lignocellulosic compounds was performed using H_2_SO_4_, glacial acetic acid (90% purity, Meyer) and sodium chlorite (80% purity, “Tensoactivos México”, Mexico City, Mexico). Finally, for the coffee pulp delignification, H_2_SO_4_, NaOH and food grade hydrogen peroxide (30% purity, Golden Bell, Edo de Mexico, Mexico) were used.

### 2.2. Physical Treatment

**Figure 1:**
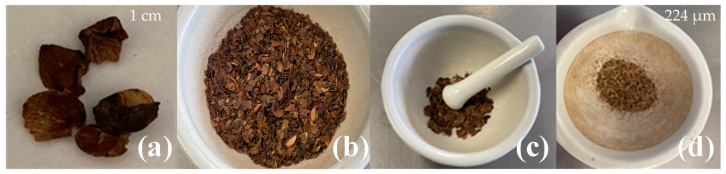
Coffee pulp size reduction: (**a**) before the physical treatment, (**b**) dehydrated, (**c**) ground and (**d**) sieved.

Figure 1 shows the physical treatment of the coffee pulp. It was submitted to a size reduction process, washed with distilled water to remove impurities and dried at 60 °C for 24 h. Once the pulp was dehydrated, it was ground in a mortar for 10 min and afterward with a Krups coffee grinder. The ground coffee pulp was sieved with Tyler sieves (MATEX, Mexico City, Mexico) with mesh sizes of 40, 60 and 80 and coupled in series for 20 min. The obtained size after the sieving process was 224 µm [28].

### 2.3. Proximate Chemical Analysis

The PChA is a physicochemical characterization that consists of establishing the nutritional value of food products by means of different techniques established by the Association of Official Agricultural Chemists (AOAC). To this end, moisture (AOAC 950.27), ash (AOAC 940.26), ethereal extracts (AOAC 920.39) and coffee pulp crude fiber (AOAC 962.09) were determined. Each analysis was performed in triplicate in order to ensure the reproducibility of the results by following the techniques proposed in Official Methods of Analysis, AOAC international [29].

### 2.4. Determination of Lignocellulosic Compounds

In order to know the composition of the lignocellulosic compounds present in the coffee pulp, the lignin (ASTM D1106), hemicellulose (ASTM D1104-56) and cellulose (ASTM D1103) contents in the coffee pulp were determined before the chemical, after the alkaline and following bleaching treatments, leading to acid hydrolysis under the norms issued by the ASTM [30,31,32]. These analyses were run in triplicate to ensure the reproducibility of the results.

### 2.5. Characterization of Coffee Pulp

After the physical treatment, the coffee pulp was characterized using the Fourier-transform infrared spectroscopy (FTIR) technique, employing an FTIR Perkin-Elmer spectrophotometer (Shelton, CT, USA). A spectrum displacement rate of 0.2 cm/s with 16 cm^−1^ resolution was applied at 8 scans per minute with a wave-number interval ranging from 400 to 4000 cm^–1^ [33]. To confirm the cellulose crystalline phase of the coffee pulp, the X-ray diffraction (XRD) technique was used by means of a Bruker model D8 Discover diffractometer (Karlsruhe, Germany), working with a 1.5418-Ǻ electron beam. The analysis conditions were carried out using CuK_α_ (5406 Å) radiation generated at 35 kV and 25 mA within a 2θ interval ranging from 5° to 70° at a rate of 0.05°/s [34]. The XRD data were analyzed using the Peakfit software version 4.11. The surface morphology of the coffee pulp was determined via scanning electron microscopy (SEM) using a JEOL model JSM—6610LV microscope (Peabody, MA, USA). The SEM observations were carried out by means of a secondary electron detector (SED) at 10 kV [35]. The processing of images to measure the diameter of the CMFs was performed by employing the ImageJ 8 software version 1.54d. The chemical qualitative analysis was achieved using energy dispersive X-ray spectroscopy (EDS) with an Oxford model INCA energy 250+ probe under electron dispersion [36].

To measure the crystallinity index (CI) of the sample, deconvolutions were performed employing Equation (1) and the average crystal size (β) was measured through the Scherrer equation, Equation (2). The obtained results were employed for measuring the crystallinity of the CMFs by means of the chemical treatments and to evaluate their effectivity by eliminating lignin and hemicellulose [37,38].

(1) $$CI\left(\%\right)=\frac{\left[I\left(1\bar{1}0\right)+I\;\left(110\right)+I\left(200\right)\right]}{I\left(1\bar{1}0\right)+I\left(110\right)+I\left(200\right)+Inoncryst)}\times 100$$

where *I*(1$\bar{1}0$), *I*(110) and *I*(200) represent the area under the crystalline plane corresponding to the 2 θ signals of 15°, 17° and 22°, respectively, and Inoncryst is the area under the crystalline peak corresponding to the diffraction pattern of amorphous cellulose.

(2) $$L=\frac{K\lambda }{\beta (\theta )cos\theta }$$

where *K* is the shape factor (0.9), *L* is the crystal average size, *λ* is the wave length of Cu radiation, *β*(*θ*) is the full width at half maximum (FWHM) and *θ* is the diffraction angle.

### 2.6. Production of Microcellulosic Fibers

Alkaline, bleaching and acid hydrolysis treatments were carried out to extract CMFs from coffee pulp and eliminate non-cellulosic compounds such as lignin, hemicellulose and pectin, among others [39]. The alkaline treatment is usually employed as a chemical pretreatment due to the saponification reaction that provokes the fragmentation of the hierarchical linking between hemicellulose and lignin for the incorporation of enzymatic or acid treatments [40]. For this reason, an experimental design consisting of a 3-variable matrix was established: NaOH concentration (10 and 20 wt.%), time (1 and 3 h) and temperature (120 and 170 °C). The development of the alkaline treatment was performed at a 1:10 ratio, employing a Soxhlet system. Afterward, the product was washed with distilled water several times until the alkaline solution was eliminated, reaching pH values between 6 and 7 [9,41].

Bleaching was used to obtain purer cellulose and to continue eliminating lignocellulosic fractions. This treatment was carried out with hydrogen peroxide at 30% with a 1:20 ratio under stirring at 50 °C for 24 h. Afterward, washing with distilled water was implemented until pH values between 6 and 7 were reached, followed by drying at 25 °C until white cellulose was obtained [33]. The bleached cellulose was submitted to acid hydrolysis with a 1:10 ratio to produce microcellulosic fibers. To this end, a H_2_SO_4_ solution at 64% was added and kept under stirring at 25 °C for 1 h. Once the reaction achieved its time, it was stopped with distilled water, the product was sedimented and the supernatant liquid was decanted. Washings took place until pH values between 6 and 7 were reached. The sediment was centrifuged at 1400 rpm for 30 min to eliminate excess acid [42,43].

## 3. Results and Discussion

### 3.1. PChA of Coffee Pulp

Table 1 shows the physicochemical characterization of the coffee pulp by PChA, observing a moisture percentage below 10%, which is related to both the time and raw material storage technique [44]. The ash value was 7.83 ± 1.08%, which is similar to the one reported by Borrelli et al. and confirmed that the minerals present in the coffee pulp are not affected by time [45]. As for the ethereal extracts, the value of 2.87 ± 0.93% was relatively low and some authors have associated it with gluten-free grains [12]. Finally, the crude fiber percentage was 18.07 ± 1.13%, thus confirming the presence of the ideal lignocellulosic compounds for the extraction of cellulose. It has been reported that the fiber percentage is directly related to the formation and ripening of the coffee fruits [27,46].

### 3.2. Characterization of the Coffee Pulp before the Chemical Treatments

Table 2 shows the percentage of the lignocellulosic compounds, which were 18.29, 20.92 and 26.06% for lignin, hemicellulose and cellulose, respectively. The obtained values are lower than those reported by Collazo et al. in their study on coffee pulp [13]. Notwithstanding, the parts corresponding to the coffee cherry and the process for obtaining the seeds can affect the lignocellulosic compositions as reported by Ballesteros et al. [39]. For instance, Reis et al. analyzed only the coffee parchment and indicated that it is rich in lignin, and its chemical composition changes depending on the analysis method and plantation region, among other factors [48].

**Figure 2:**
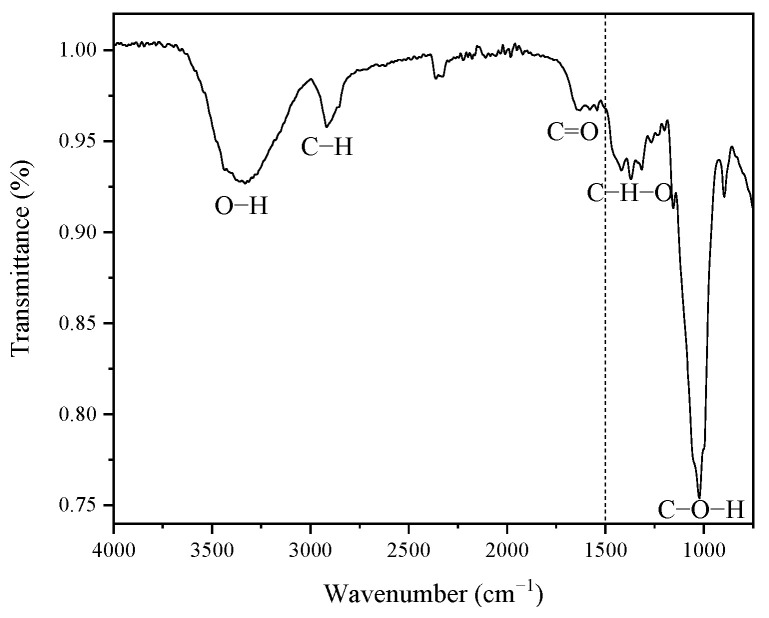
FT-IR spectrum of the coffee pulp before the chemical treatments.

Figure 2 shows the functional groups present in the coffee pulp before the chemical treatments, displaying five signals ranging from 1500 cm^−1^ to 4000 cm^−1^. The high region (1500–4000 cm^−1^) reveals a signal located at 3291.77 cm^−1^, which corresponds to the stretching vibration of the O-H bond of the hydroxyl group, which confirms the hydrophilic characteristic of the coffee pulp cellulose [49].

A second signal is located at 2922.51 cm^−1^, which belongs to the stretching vibration of the C-H bond, and to alkyl groups of lignin aliphatic bonds, hemicellulose and cellulose, where carbon has sp^3^ hybridization [34]. The signal with a width narrower than the first one, found at 1728.50 cm^−1^, corresponds to the bond stretching of C=O and is characteristic of carboxyl groups, which confirms the presence of the lignin ferulic and p-coumaric compounds and hemicellulose xylan acetyl [39,49]. As for the low region (400–1500 cm^−1^), the signals associated with the wave numbers 1375.41 cm^−1^ and 1316.16 cm^−1^ were assigned to the C-H-O bending [50]. The most intense signal was identified at 1027 cm^−1^, which was ascribed to the stretching vibration of C-O-H, which corresponds to the structure of the cellulosic compound [33].

**Figure 3:**
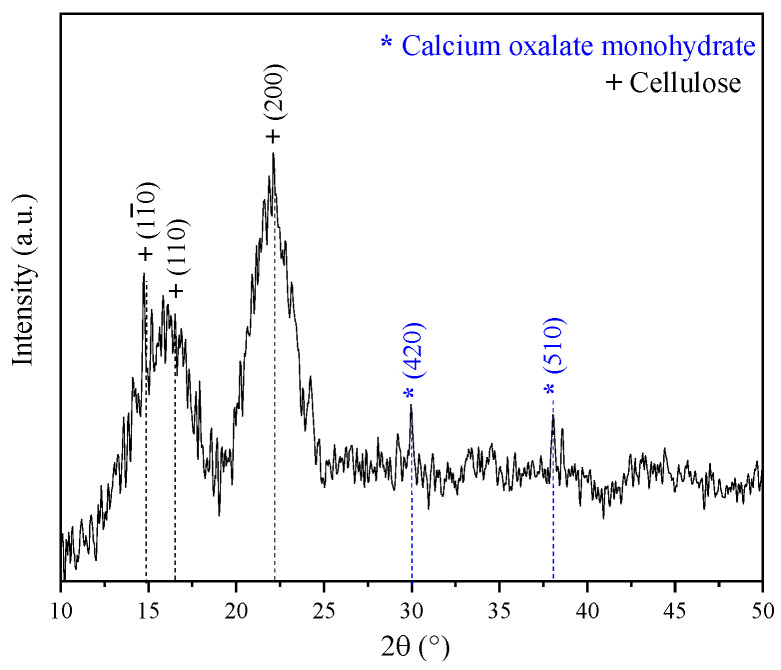
XRD pattern of the coffee pulp before the chemical treatments.

Figure 3 displays the XDR pattern of the coffee pulp before the chemical treatments. The signals associated with the Bragg angles at 15°, 17° and 22° in 2θ are related to Type I cellulose reflections (PDF 56-1719) [34]. This result is in good agreement with the XRD pattern shown by Collazo et al. who analyzed coffee pulp and obtained signals at 15°, 16° and 22° in 2θ, reporting that these diffraction peaks match those of Type I cellulose [13]. Also, peaks at 30° and 37° in 2θ were observed, which corresponded to calcium oxalate (PDF 01-0157). The Ca signal in the XDR pattern spectrum can be associated with the presence of calcium oxalate, characteristic of vascular plants that synthesize glucose through CO_2_ fixation [51].

**Figure 4:**
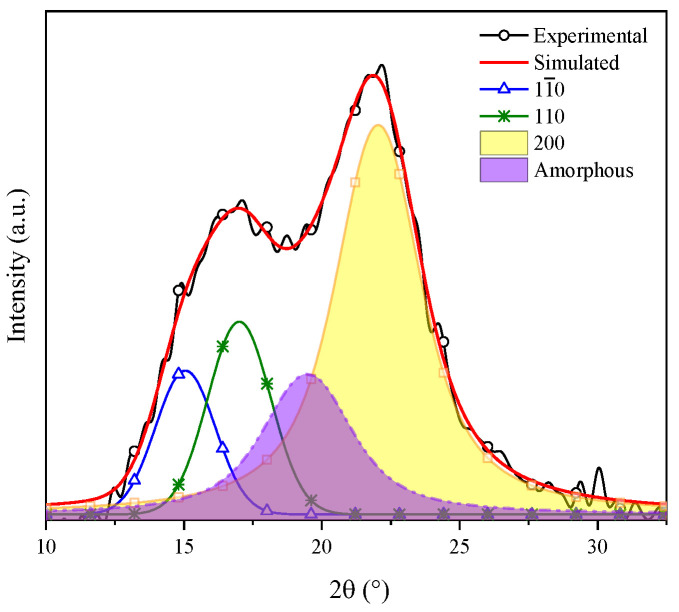
Deconvolution of the XRD pattern of cellulose present in the coffee pulp before the chemical treatments.

Gong et al. indicated that cellulose is a polyphorm material and that its transformations are related to either crystallinity increase or decrease. Then, the crystallinity index (CI) grows as the lignin and hemicellulose fractions are eliminated, for these are amorphous compounds [52]. The CI was determined by means of the deconvolution method through Gaussian functions. Figure 4 shows the XRD pattern of coffee pulp before the chemical treatments, where the area of the most intense signal corresponds to the peak at 22°, ascribed to the (200) plane, and the amorphous area is associated with the angle at 19.3° of Type I cellulose. The CI of this sample was 57.14%, which confirmed the crystalline phase in the sample. The obtained value is higher than the one reported by Reis et al. who employed coffee parchment with a CI of 50.6% [48].

**Figure 5:**
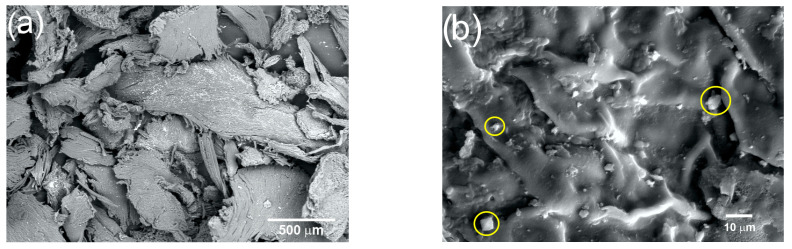
Micrograph of the coffee pulp at (**a**) 50× and (**b**) 1000× (calcium oxalate crystals marked with yellow circles).

The changes undergone by the coffee pulp fibers obtained with the physical treatments were analyzed using SEM. Figure 5a corresponds to the micrograph of coffee pulp at 50× after the physical treatment, where irregular clusters of cellulosic fibers, due to the presence of lignin and hemicellulose, can be observed. Figure 5b (1000×) shows coffee pulp irregular topography, characterized by joints between fibers, which have orientations that obey the hierarchy of lignocellulosic compounds [48]. In addition, the presence of calcium oxalate crystals (marked with yellow circles), with an average size between 10 and 15 µm and confirmed via EDS and XRD, can be observed [51].

The identification of the main coffee pulp compounds before the chemical treatments was performed using EDS. The results indicated that the prevailing elements were carbon and oxygen, with 43.97 and 52.96 wt.%, respectively, which are evidently mostly part of the cellulosic material matrix. Furthermore, Ca was identified at 1.97%, which was ascribed to the presence of calcium oxalate. At a lower proportion, Na and Mg were identified at 0.47 and 0.65%, respectively, which are characteristic elements of crude fiber impurities.

### 3.3. Characterization of the Coffee Pulp after the Chemical Treatments

Table 3 shows the lignocellulosic content with different NaOH concentrations and temperatures established in the experimental design.

**Figure 6:**
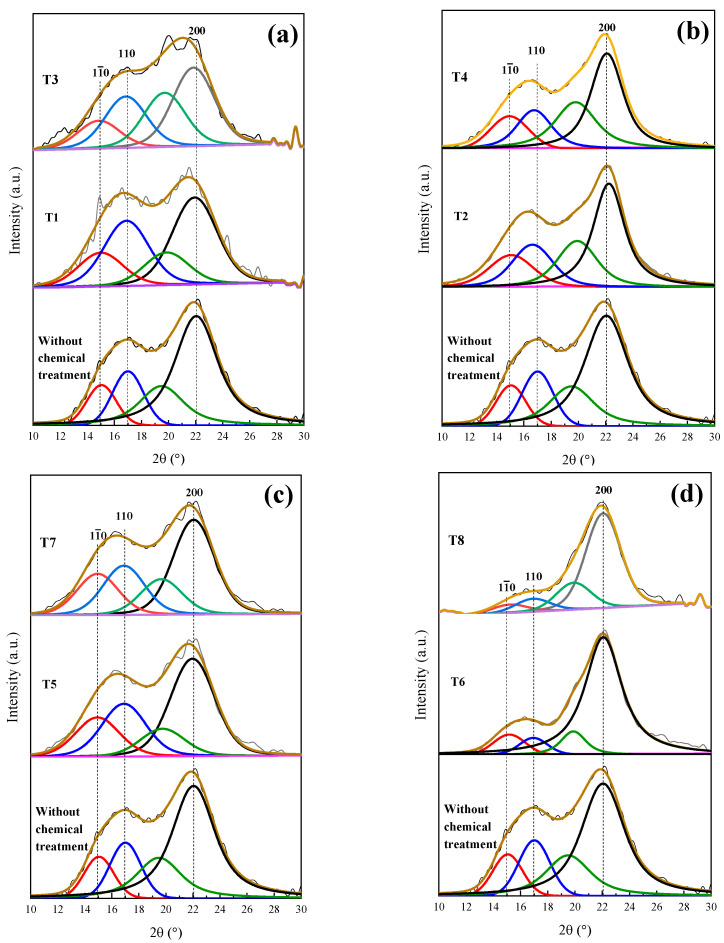
Deconvolution of XRD patterns of the coffee pulp: (**a**) temperature variation, (**b**) concentration variation, (**c**) time variation and (**d**) optimal conditions. Line black, blue, red and green correspond to diffraction index of cellulose type 1 (200), (110), (1$\bar{1}$0) and amorphous compounds, respectively.

T1 and T3 show deficient delignification, for lignin was reduced by 0.71 and 1.95%, respectively. The employed methods, when obtaining a cellulosic material, depend on the material cellulose, its pretreatment and its disintegration process. Figure 6a presents the XRD patterns of T1 and T3, preserving the angles at 15°, 17° and 22° in 2θ, corresponding to Type I cellulose and evidencing that the amorphous character of the T1 and T3 samples was not modified.

In Table 3, it is observed that T2 and T4 had cellulose increases of 8.64 and 14.2%, respectively, and lignin reductions of 2.8 and 2.96%, respectively. Wijaya et al. worked at alkaline concentrations of 20% for bamboo sprouts and obtained optimal results. Despite these results being satisfactory, it is still possible to reduce the lignin and hemicellulose percentages. Figure 6b displays the T2 and T4 XRD patterns, where a sharper peak for T4 at 22° in 2θ can be observed, which confirms the reduction in the cellulose amorphous phase [9]. It is worth emphasizing that the T1 and T3 treatments were carried out under the same conditions, but with higher NaOH (20%) concentrations, which suggests that such NaOH increase improved the efficiency of the delignification process.

The 3 h alkaline treatments reached lower lignin values, which is the case for T5 and T7, which demonstrated delignification at 1.97 and 3.47%, respectively, which are similar to the percentages obtained with T2 and T4, as shown in Table 3. Dai et al. implemented a time of 10 h and concentration of 10%, performing the total removal of lignin [53]. This result implies that the time taken for alkaline digestion provokes the resistance of the biomass chemical structure to weaken temporarily. In Figure 6c, the XRD patterns of the original sample (T5 and T7) are displayed, where changes in the intensities of the signals at 15°, 17° and 22° in 2θ, characteristic of Type I cellulose, can be observed.

The best results were possible with T6 and T8, where the increases in cellulose percentage were 22.68 and 24.11%, respectively. The last treatment took place at 170 °C, as shown in Table 3, which confirmed that temperature is a decisive factor during the delignification process. This fact can be verified with T7, which was carried out at 10% of NaOH for 3 h at 170 °C, giving a cellulose yield of 15%. These results were confirmed by the XRD analysis featured in Figure 6d. As for T8, the intensity of peaks located at 15 and 17° decreased, but the signal at 22° in 2θ was preserved [15]. Due to the aforementioned results, it can be concluded that T6, which was performed at 20% of NaOH and 120 °C for 3 h, achieved the best results regarding the elimination of the amorphous phase and presented a diffraction pattern characteristic of the crystalline phase of cellulose Type I. Johar et al. worked with rice husk to obtain nanocellulose, finding that with alkaline treatment, the stiffness of the cellulosic fibers increased, thus concluding that lignin and hemicellulose removal had occurred [33].

**Figure 7:**
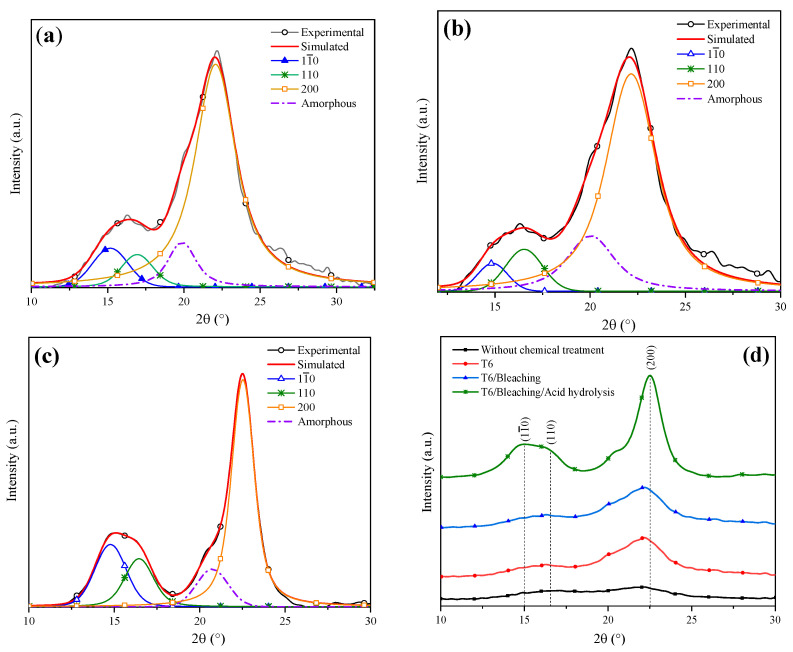
Deconvolution of XRD patterns: (**a**) T6, (**b**) bleaching treatment, (**c**) acid hydrolysis and (**d**) comparison of the chemical treatments.

Figure 7a shows the deconvolution of the T6 XRD pattern through which a CI of 75% was determined. Dai et al. analyzed pineapple crowns at a NaOH concentration of 10% and obtained a CI difference of 11% with respect to their original sample [53]. Conversely, Carrión Prieto et al. worked with a NaOH concentration of 25% at room temperature and obtained a CI increase of 12% in comparison with their sample without treatment [54]. Based on the previous points, it is possible to conclude that the factors established in the experimental design improved the cellulose yield. The T6 average crystal size was 2.915 nm (Table 4 ), indicating that a NaOH concentration of 20% reduced the lignin and hemicellulose content. It has been reported that the cellulose crystal size growth is due to the widening of the length of the polydispersed and crystalline columns [55]. Similar results were reported by Jin et al. who indicated that with NaOH alkaline treatment, an average crystal size of 2.8 nm was obtained [15].

Figure 7b features the deconvolution of the XRD pattern of the T6 sample after applying the bleaching agent. A CI of 80% was obtained, indicating that the cellulose content was increased. Kalita et al. obtained cellulose fibers from rice husk using a bleaching mixture of sodium hydroxide, acetic acid and sodium hypochlorite solution, which helped to reach a cellulose yield of 52% [56]. In order to enhance the cellulose yield even more, an acid hydrolysis process was performed. The sample XRD analysis after this treatment is shown in Figure 7c, from which a CI of 94% was determined. Table 4 shows the lignocellulosic composition of the coffee pulp sample after the acid hydrolysis treatment, where a cellulose increase of 0.7% was observed. Similar results were reported by Collazo et al. who indicated CI increments from 52 to 94% and a cellulose growth of 0.9% after applying acid hydrolysis [13].

Figure 7d displays the XRD patterns after the application of different chemical treatments. It is observed that with the bleaching and acid hydrolysis treatment, the removal of the amorphous fraction was higher and preserved the diffraction pattern of Type I cellulose at the angles 15°, 17° and 22° in 2θ, revealing sharper and more intense peaks after the last stages of chemical treatments.

**Figure 8:**
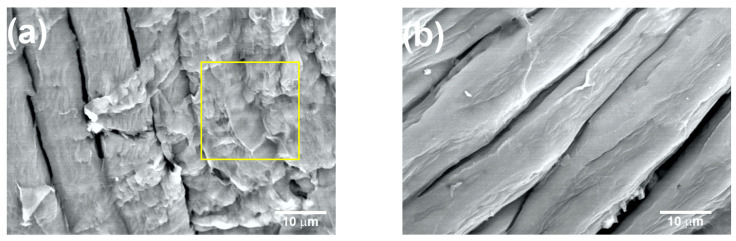
SEM micrographs at 2000× of the coffee pulp after the alkaline treatments: (**a**) T5 and (**b**) T6. Lignin and hemicellulose fractions marked with yellow square.

Figure 8a shows the coffee pulp surface after T5 (NaOH at 10%, 120 °C and 3 h). The characteristic morphological orientation of the CMFs can be observed, for which the average diameter was 10 ± 2 µm. Also, a lower amount of the lignin and hemicellulose fractions can be appreciated (yellow square). As seen in the micrograph, with T5, lignin and hemicellulose percentage reduction was achieved, but the formation of the CMFs was not complete. Similar micrographs were obtained for biomass lignin by C. Landanifertallo [54]. Figure 8b corresponds to coffee pulp after T6 (NaOH at 20%, 120 °C and 3 h). Higher degradation of the lignocellulosic fraction can be observed, obtaining CMF with a diameter of 8 ± 2 µm. Furthermore, the polymerization degree diminished through a depolymerization process as a consequence of the alkaline treatment with NaOH at 20%, thus confirming that this alkaline process is more efficient. The EDS analysis of the T6 sample revealed an increase in the elements carbon (45.23 wt.%) and oxygen (54.65 wt.%) with respect to the sample before the chemical treatment, which confirmed the presence of cellulose. In a study carried out by Wijaya et al. using bamboo cellulose treated with NaOH at 20% under conditions similar to those employed here, the achievement of well-defined CMFs was reported [57].

**Figure 9:**
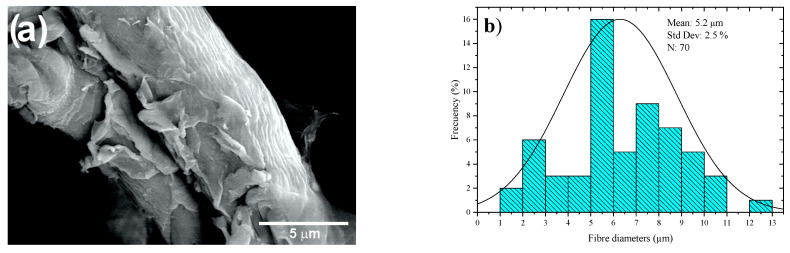
(**a**) SEM micrographs at 3000× and (**b**) histogram of the diameter distribution of the coffee pulp CMFs after the alkaline treatment, bleaching and acid hydrolysis.

Figure 9a shows SEM images of the CMFs of coffee pulp after T6, bleaching and acid hydrolysis. Isolated microfibers are observed due to the action of the oxidizing agent (H_2_O_2_) and acid hydrolysis. Figure 9b features a histogram of the diameter distribution of the coffee pulp CMFs after the chemical treatments, indicating that the average diameter of the CMFs was 5.2 ± 2.5 µm, which was possible due to the combination of chemical treatments, which can give higher resistance to the polymeric matrices [58]. This methodology for cellulose extraction has been employed for obtaining individual CMFs from different biomass sources with results similar to those reported elsewhere [59].

## 4. Conclusions

The coffee pulp employed in the present research work presented a composition of 8.32% of moisture, 7.83% of minerals, 2.87% of fat and oils and 18.07% of crude fiber. With this crude fiber percentage, it was confirmed that it is possible to obtain cellulose from lignocellulosic compounds having 18.29% of lignin, 20.92% of hemicellulose and 26.06% of cellulose. These results were supported by the FTIR analysis, which revealed a signal within the region ranging from 3000 cm^−1^ to 3500 cm^−1^, characteristic of the cellulose OH groups; the C-H and C=O signals in the region from 1500 cm^−1^ to 3000 cm^−1^ correspond to lignin and hemicellulose; and, finally, the signals within the 400–1500 cm^−1^ region stemming from the C-H-O stretching were attributed to lignin and cellulose. As for the cellulose separation, it was observed that the alkaline treatment T6, which employed NaOH at 20%, 120 °C and 3 h, presented better results with lignin, hemicellulose and cellulose contents of 13.59, 15.49 and 48.74%, respectively. By means of bleaching and acid hydrolysis, CMFs of 5 ± 2 µm were obtained with lignin, hemicellulose and cellulose percentages of 0.05, 0.07 and 64.7%, respectively. The CIs of the coffee pulp samples were 57.14%, 75% (alkaline treatment), 80% (bleaching) and 94% (acid hydrolysis). The final average crystal size was 3.854 nm. The solid organic residue produced during the transformation of coffee becomes a raw material source for obtaining CMFs to produce green materials.

## Figures and Tables

**Table 1:** Physicochemical characterization of the coffee pulp by PChA.

Compounds	Experimentation	Borrelli et al. [45]	Pourfarzad et al. [47]
Moisture %	8.32 ± 1.04	7.30	7.1
Ash %	7.83 ± 1.08	7.0	7
Ethereal extracts %	2.87 ± 0.93	2.2	2.2
Crude fiber %	18.07 ± 1.13	53.7	53

**Table 2:** Lignocellulosic compounds of the coffee pulp.

Lignocellulosic Compounds of Coffee Pulp	Experimentation	Ballesteros et al. [39]	Reis et al. [48]	Collazo et al. [13]
Lignin %	18.29 ± 0.98	23.90	53	23.2
Hemicellulose %	20.92 ± 1.49	12.04	18	18.2
Cellulose %	26.06 ± 0.83	39–10	22	35.4

**Table 3:** Lignocellulosic content with different alkaline treatments.

Treatment (T)	NaOH Concentration (wt.%)	Temperature (°C)	Time (h)	Lignin (%)	Cellulose (%)	Hemicellulose (%)
Without treatment	-	-	-	18.29 ± 0.98	26.06 ± 0.83	20.92 ± 1.49
1	10	120	1	17.58 ± 1.52	28.16 ± 1.59	19.22 ± 1.08
2	20	120	1	15.49 ± 1.43	34.70 ± 1.29	18.11 ± 1.78
3	10	170	1	16.34 ± 1.97	29.92 ± 0.42	19.27 ± 0.67
4	20	170	1	15.33 ± 1.14	40.26 ± 0.99	17.47 ± 0.67
5	10	120	3	15.61 ± 1.70	37.92 ± 1.61	16.97 ± 1.68
6	20	120	3	13.58 ± 1.28	48.74 ± 0.53	15.49 ± 1.06
7	10	170	3	14.82 ± 1.35	40.69 ± 1.46	13.05 ± 1.22
8	20	170	3	13.64 ± 1.50	50.17 ± 1.78	13.64 ± 1.05

**Table 4:** Lignocellulosic composition of coffee pulp after different chemical treatments.

Component	Lignin (%)	Cellulose (%)	Hemicellulose (%)	Crystallinity Index (%)	Average Crystal Size (nm)
Coffee pulp	18.29	26.06	20.92	57.14	2.16
T6	13.58	48.74	15.49	75.42	2.91
Bleaching	1.07	57.5	2.37	80.12	3.41
Acid hydrolysis	0.05	64.7	0.07	94.67	3.85

## Data Availability

The data presented in this study are available on request from the corresponding author.
